# The research development of STAT3 in hepatic ischemia-reperfusion injury

**DOI:** 10.3389/fimmu.2023.1066222

**Published:** 2023-01-24

**Authors:** Hanwen Yang, Pengpeng Zhang, Qiang Wang, Ke Cheng, Yujun Zhao

**Affiliations:** Engineering and Technology Research Center for Transplantation Medicine of National Health Comission, Third Xiangya Hospital, Central South University, Changsha, China

**Keywords:** signal transducers and activators of transcription3 (STAT3), ischemia-reperfusion injury (IRI), mitochondria, cell apoptosis, liver

## Abstract

Ischemia-reperfusion injury (IRI) is a common complication of surgery, which can cause rapid deterioration of the liver function, increase the risk of graft rejection, and seriously affect the prognosis of patients. The signal transducer and activator of transcription 3 (STAT3) protein has been implicated in pathogenesis of IRI. STAT3 influences the mitochondria through multiple pathways and is also involved in apoptosis and other forms of programmed cell death. STAT3 is associated with Janus kinase (JAK), phosphoinositide-3 kinase (PI3K), and heme oxygenase-1 (HO-1) in liver IRI. The STAT3 pathway plays a dual role in IRI as it can also regulate lipid metabolism which may have potential for treating IRI fatty liver. In this review, we summarize research on the function of STAT3 in liver IRI to provide references for its application in the clinic.

Ischemia-reperfusion injury (IRI) is a common complication after liver surgery (such as surgery for liver cancer or liver transplantation). The interruption of the oxygen supply during ischemia causes hepatic sinusoidal stenosis as well as secondary microcirculation disorders (1, 2). Various factors such as tissue hypoxia, nutrient deficiencies, and metabolic disruption during ischemia can lead to hepatocyte injury. Inflammatory factors, apoptotic pathways, and reactive oxygen species (ROS), which are activated during reperfusion, can result in a rapid deterioration of the liver function, which also increases the risk of rejection and can adversely affect patient prognosis (3, 4). IRI is usually classified as warm IRI *in vivo* and cold IRI *in vitro*. Although both are primarily caused by hypoxia and the consumption of substrates caused by ischemia, the treatment methods differ due to the differences in the temperature and cell metabolic energy (5). Liver IRI is still a major problem in liver surgery, and no effective treatments are currently available. Signal transducer and activator of transcription (STAT) proteins are a class of transcription factors present in the cytoplasm, and mainly function to transmit signals from cell-surface receptors to the nucleus. The STAT family consists of seven distinct members, namely, STAT1, STAT2, STAT3, STAT4, STAT5A, STAT5B, and STAT 6. These STAT proteins contain between 750 and 850 amino acids and have similar structures and functions. STAT3 is composed of six different functional regions, namely, the N-domain/STAT protein interaction domain, coiled-coil domain (CCD), DNA-binding domain (DBD), linker domain, the SH2 domain, and the Transcriptional Activation Domain (TAD) (6, 7). There are prior reviews about the involvement of STAT3 in many organs. However, the role of STAT3-related signaling pathways in liver IRI has not been systematically summarized. Therefore, we have analyzed the relevant literature with keywords such as STAT3, ischemia reperfusion injury, and liver, amongst others. This paper aimed to review the various studies of STAT3 in liver IRI and discuss its associated pathways and different roles, to provide a reference for further research.

## 1 Structure and function of STAT3 and the relationship between STAT3 and the mitochondria

The human STAT3 gene is located on chromosome 17q21 and encodes an 89-kDa protein (8). STAT3 mainly consists of three different isoforms, namely STAT3α, STAT3β, and STAT3γ, of which the first is the most common. STAT3α can bind to IL-6 and IL-10 secreted by macrophages. STAT3β can inhibit the synthesis of inflammatory factors and plays an anti-inflammatory role while STAT3γ is mainly produced by the degradation of STAT3α and is activated by differentiated neutrophils (9, 10).

**Figure 1:**
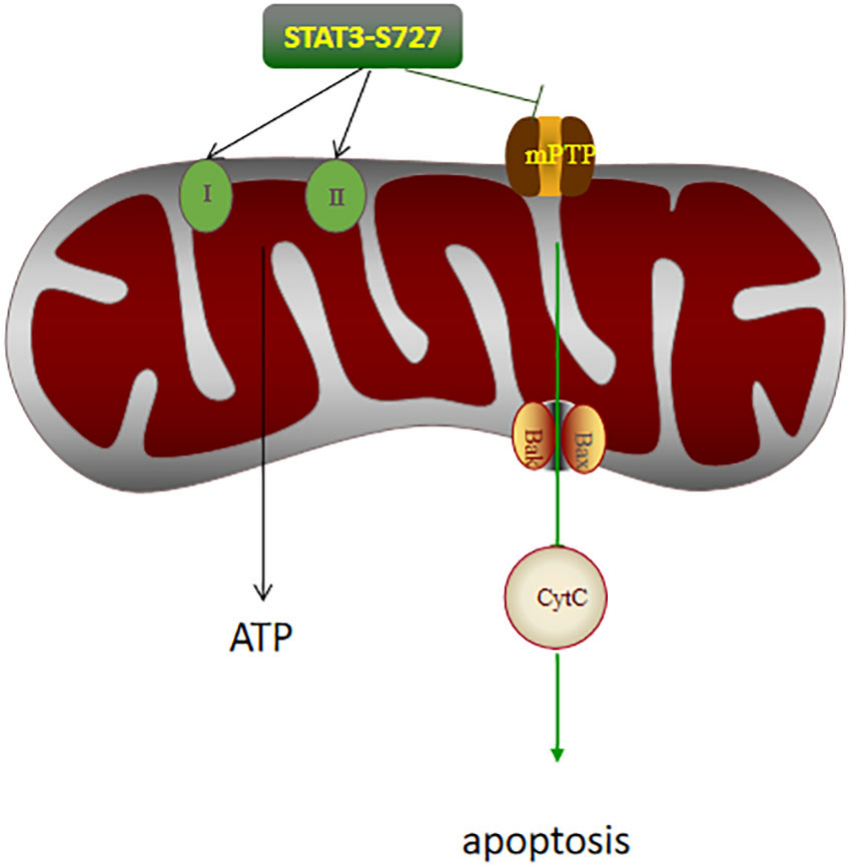
The effects of P-STAT3 on the mitochondria in hepatic IRI. P-STAT3 can promote ATP synthesis by increasing the activity of respiratory chain complex I and II. P-STAT3 also inhibited the opening of mPTP, thereby inhibiting the expression of Bax and the release of CytC, and finally alleviating apoptosis.

Moreover, based on *in vitro* and *in vivo* experiments, Lucy Xi Lou showed that STAT3 knockout resulted in increased expression of transaminase and inflammatory indicators suggesting that endogenous STAT3 plays a protective role in IRI (11). However, the upstream and downstream pathways associated with STAT3 were not investigated in this study, and the specific mechanism requires confirmation by subsequent experiments. Endogenous negative regulators of STAT3, such as suppressor of cytokine-induced STAT signaling (SOCs), can bind to activated receptors and interact with Janus kinase (JAK), which in turn inhibits the activation of the STAT pathway (12). In addition, there are nuclear factors that can bind to phosphorylated STAT, commonly known as PIAS (protein inhibitors of activated STATs), of which PIAS3 is a specific inhibitor of STAT3. It can block dimerization of the STAT3 monomer or promote the dissociation of dimerized STAT3, thus inhibiting STAT3 activation (13). STAT3 has two different phosphorylation sites, namely, Tyrosine 705 (Y705) and Serine 727 (S727). STAT3 phosphorylated at Y705 dimerizes and translocates to the nucleus, while phosphorylation at S727 leads to translocation to the mitochondria (14, 15). P-STAT3 regulates the activity of the electron transport chain (ETC) through S727 (16). Mitochondria are the main sites for ROS production, and the ETC is the most important source of ATP (17). In IR, excessive ROS and Ca^2+^ can cause the opening of the mitochondrial permeability transfer pore (MPTP) and adversely affect the mitochondrial membrane potential. This, in turn, can lead to peroxidation of the mitochondrial membrane, the release of cytochrome c, the inhibition of ATP synthesis, and, finally, irreversible cell death caused by mitochondrial membrane peroxidation (18, 19). ROS can activate STAT3 during IRI (20). STAT3 can inhibit MPTP opening caused by ROS production (**Figure 1**), thereby reducing mitochondrial damage (21). The levels of P-STAT3 in the mitochondria increase rapidly during reperfusion, while the P-STAT3 level in the cytosol decreases rapidly (22). Phosphorylated STAT3 (P-STAT3) is usually present in mitochondrial inner membrane adjacent to the matrix and is important for maintaining mitochondrial integrity. GRIM-19, the main component of mitochondrial complex I, promotes the entry of P-STAT3 into the mitochondria (23).The binding of P-STAT3 to the respiratory chain increases the membrane potential and increases ATP production. STAT3 knockdown can inhibit the rate of mitochondrial respiratory chain and complex I, II activity, which can then lead to the release of excess cytochrome C, thereby aggravating IRI (24).

## 2 STAT3-related modes of death

Liver IRI is associated with various forms of cell death, including necrosis, apoptosis, autophagy, and ferroptosis, but those most associated with are apoptosis and autophagy. Apoptosis is a form of programmed cell death responsible for the maintenance of homeostasis in multicellular organisms (25). A number of studies have reported that 50-70% of endothelial cells and 40-60% of hepatocytes undergo apoptosis during reperfusion (26, 27). STAT3 can inhibit apoptosis in two distinct ways. First, STAT3 can play a direct anti-apoptotic role by upregulating the expression of the anti-apoptotic protein Bcl-2 and downregulating the expression of the pro-apoptotic protein Bax (28–30). Second, STAT3 can also inhibit MPTP formation to stabilize the mitochondrial membrane potential ΔΨm and thereby reduce ROS production, both of which can simultaneously inhibit the release of apoptosis-related cytokines, suppress caspase-related death pathways, attenuate the fragmentation of genetic material DNA, and ultimately inhibit apoptosis (31, 32). Autophagy is a process involved in the degradation of proteins and organelles in cells. Autophagy-related 5 (ATG5) and Microtubule-associated protein 1 Light BII (LC3BII) are two important autophagy-related proteins in IR. Yufang Han found that STAT3 was able to activate ATG5-mediated autophagy, thereby attenuating IRI (33). Shipeng Li reported that microRNA-17 (mir-17) promoted the expression of autophagy protein LC3BII by inhibiting the expression of STAT3, and ultimately aggravated liver IRI (34). Therefore, the potential relationship between STAT3 and liver IRI reported during autophagy needs to be further explored.

## 3 STAT3 and liver cells

The liver is the largest parenchymal organ in the human body, and contains non-inflammatory cells such as hepatocytes and endothelial cells as well as inflammatory cells such as Kupffer cells and lymphocytes.

### 3.1 STAT3 and non-inflammatory cells

Hepatocytes account for 80% of the liver tissue maintaining its main metabolic functions. In the carbon tetrachloride and alcohol models of acute liver injury, the inflammatory index was found to be lower in STAT3-knockout mice, while the inflammatory index was higher in the ConA-induced hepatitis and LPS-induced models of STAT3-knockout mice. STAT3 may inhibit inflammation by inhibiting STAT1, so STAT3 can inhibit the activation of the pro-inflammatory factor STAT1 in ConA-induced and LPS-induced hepatitis (35–38). STAT3 has a dual role in hepatocytes. Model differences are one of the important reasons, which need to be further explored in other models in the future. Some researchers have found that the degree of apoptosis and increased inflammatory response in mice with endothelial-cell STAT3 knockout in the alcoholic liver model, but the specific mechanism has not been explored. There are few studies on STAT3 in endothelial cells, and further exploration of its actions is needed in the future (39).

### 3.2 STAT3 and inflammatory cells

**Figure 2:**
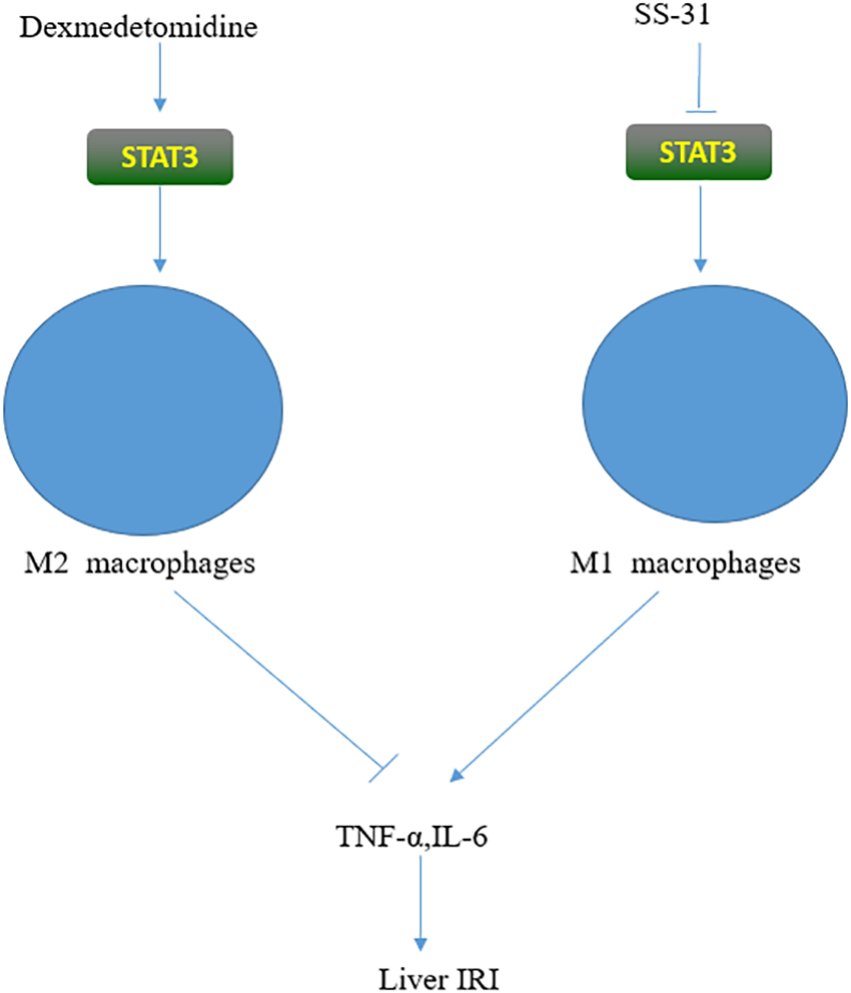
STAT3 has opposing effects on macrophages. On the one hand, it can promote the release of inflammatory factors and aggravate IRI by activating M1; on the other hand, it can activate M2, inhibit the release of inflammatory factors, thus alleviating IRI. The different effect depends on the activator of STAT3.

Hepatic macrophages are termed Kupffer cells (KCs). KCs account for 20% to 35% of all the non-parenchymal cells in the liver and are an important component of the immune cell compartment. KCs can generate oxidative stress through regulating different pathways and stimulating the production of TNF-α and other inflammatory factors, thereby aggravating IRI (40). KCs act mainly through the recognition of Toll-like receptors (TLRs), which are important receptors involved in the inflammatory cascade (41, 42). TLR4 is the most important member of the TLR family, and STAT3 is one of its important ligands. TLR4-deficient mice have significantly reduced IRI. KCs are activated by two mechanisms (**Figure 2**), M1 and M2. M1 can release various inflammatory factors and cause tissue damage, whereas M2, in contrast to M1, has anti-inflammatory effects (43, 44). SS-31 is a novel antioxidant targeting mitochondria, whose main effects include promoting the production of ATP and inhibiting ROS production (45, 46). Longcheng Shang reported that SS-31 could inhibit the production of mitochondrial ROS, thereby reducing the phosphorylation of STAT1 and STAT3. This can suppress the polarization of M1 macrophages, inhibit the release of inflammatory factors such as TNF-α and IL-1β, and ultimately alleviate liver IRI (47). Dexmedetomidine is a selective α2 adrenergic receptor agonist used for sedation and anesthesia in surgical patients. Haoming Zhou found that dexmedetomidine could activate the peroxisome proliferator-activated receptor-γ (PPARγ)/STAT3 pathway, thereby promoting the activation of M2 macrophages, suppressing the release of TNF-α and other inflammatory factors, and ultimately alleviating liver IRI (48–50). Zhuqing Rao reported that hyperglycemia could aggravate liver IRI by inhibiting the polarization of M2 macrophages and IL-10 activation by inhibiting STAT3 through CCAAT/enhancer-binding protein(C/EBP) protein-mediated ER stress (51). Roquin-1 is an E3 ubiquitin ligase originally identified in a mutated gene in SLE mice (52). Lei Zheng found that Roquin-1 effectively inhibited the polarization of M1 macrophages and promoted the activation of M2 macrophages, which inhibited AMP-activated protein kinase a (AMPKa) activity and promoted the activation of mammalian target of rapamycin (mTOR) and STAT3, which, in turn, led to the reduced production of related inflammatory factors and ultimately alleviated hepatic IRI (53). Tammy M found that proteolysis inducing factor (PIF) may activate STAT3 in human Kupffer cells, thereby inducing the inflammatory response. To improve the condition of patients with cachexia, inhibitors of this pathway should be further investigated (54). Lara Campana found that the STAT3-IL10-IL6 pathway promotes phenotypic transformation of human macrophages, thereby alleviating acute liver injury (55). Ozturk Akcora STAT3 inhibitor BWP1066 inhibits the release of inflammatory cytokines from human macrophages, thereby alleviating acute liver injury (56). STAT3 is essential for the growth and development of B lymphocytes, and IL-21 secreted by T cells promotes the transformation of CD19+B cell precursors into plasma cells that secrete IgG. Leptin can promote the secretion of IL-6 and TNF-α by human B cells through activation of the JAK2/STAT3 pathway and thus aggravate the inflammatory response (57–59). STAT3 in CD8+T lymphocytes is closely related to IL-21. In CD8+T lymphocytes, IL-6 can promote the expression of STAT3, which promotes the production of IL-21 and ultimately stimulates the production of CD8+ memory cells (60). STAT3 plays an important role in Th17 cells. STAT3 promoted the secretion of the anti-inflammatory factors TGF-β1 and IL-10 by CD4+T lymphocytes, inducing more Th3 cells (61).

## 4 STAT3 and upstream inflammatory cytokines

KCs can secrete several inflammatory cytokines. Many inflammatory factors such as the interleukin family (ILs) can act as ligands to influence STAT3 activation. ILs that function as ligands mainly include IL-6, IL-11, and IL-22. For example, Heng Zhou reported that after vagotomy, the expression of IL-22 was decreased, and then the expression of STAT3 was reduced, inflammatory cell infiltration was increased, and IRI was aggravated. Exogenous IL-22 supplementation can promote the phosphorylation of STAT3, thereby promoting the expression of the cyclin D1 gene, and ultimately reversing liver IRI; however the mechanisms through which cyclin D1 can potentially reverse IRI in this model needs to be further explored (62). Paul J Chestovich found that IL-22 could effectively promote the phosphorylation of STAT3, inhibit the production of inflammatory factors, and ultimately reduce liver IRI but the endogenous IL-22 content was significantly increased after 24 hours of reperfusion (63). Bai, Y reported that IL-22 could activate STAT3, inhibit apoptosis and oxidative stress, and alleviate biliary IRI after liver transplantation (64). Wanzhen Li found that Ac2-26, a derivative of the endogenous inflammatory inhibitor Annexin A1 (AnxA1), can inhibit hepatocyte apoptosis induced by the mitochondrial pathway through activation of the IL-22/STAT3 pathway. Ac2-26 can also protect ATP and the mitochondrial membrane potential (MMP), inhibit MDA and ROS production, thus reducing IRI (65, 66). Nicolas Melin found that the TLR5 agonist CBLB502 can attenuate hepatic IRI by binding to the TLR5 receptor and stimulating IL-22 production by affecting the different immune cells through activation of STAT3 (67). These findings suggest that IL-22 can act as an important inflammatory factor and associate closely with STAT3, which is worthy of further study in the future. Miao Zhu showed that IL-11 could inhibit the phosphorylation of STAT3, thereby suppressing the activation of inflammatory factors such as TNF-α and IL-10, and thus attenuating liver IRI (68). Some relevant inflammatory factors play an important role in chronic liver injury, and IL-17A is a key factor in liver fibrosis. Xiao Wei Zhang found that activation of the IL-17A/STAT3 pathway can inhibit autophagy in liver cells, thus aggravating liver fibrosis, while an IL-17A inhibitor could reverse the development of fibrosis (69). Hongwei Tang added IL-6 rs1800796 into human L02 cells to activate the IL-6/STAT3 pathway, inhibit the expression of autophagy proteins, and thus reduce IRI. Recombinant human IL-6 can be a therapeutic target for hepatic IRI (70). Kun Xie found that exosomal mir-1246 derived from human umbilical cord blood mesenchymal stem cells can regulate the balance of helper/modulator T cells through the mir-1246-mediated IL-6-gp130 (IL-6 receptor)-STAT3 axis, which ultimately could attenuate liver IRI (71). Matsumoto pointed out that ischemic preconditioning (IPC) can significantly reduce hepatic IRI through activation of the IL-6-GP130-STAT3 axis, but the specific mechanisms require further exploration (72). Dayoub R found that the IL-6-STAT3-thrombopoietin (TPO) pathway can stimulate the production of megakaryocytes in the spleen and bone marrow and play a hemostatic role after acute liver injury (73). Rania Dayoub found that exogenous the acute phase response (ALR) can inhibit the IL-6/STAT3 pathway in L-02 cells, inhibit acute phase proteins (APPs) and thus ultimately inhibit inflammatory response. However, endogenous ALR activates the IL-6/STAT3 pathway and enhances the inflammatory response, so ALR has a dual role. However, the relationship between ALR and STAT3 phosphorylation needs further investigation (74). IL-6 is pleiotropic factor. As a ligand, it can play a positive role by activating the various downstream proteins. On the other hand, persistent release of IL-6 in has been implicated in various diseases. Thus, the key challenge remains to effectively balance the physiological and pathological functions of IL-6 in cells.

## 5 JAK - STAT3 pathway

**Figure 3:**
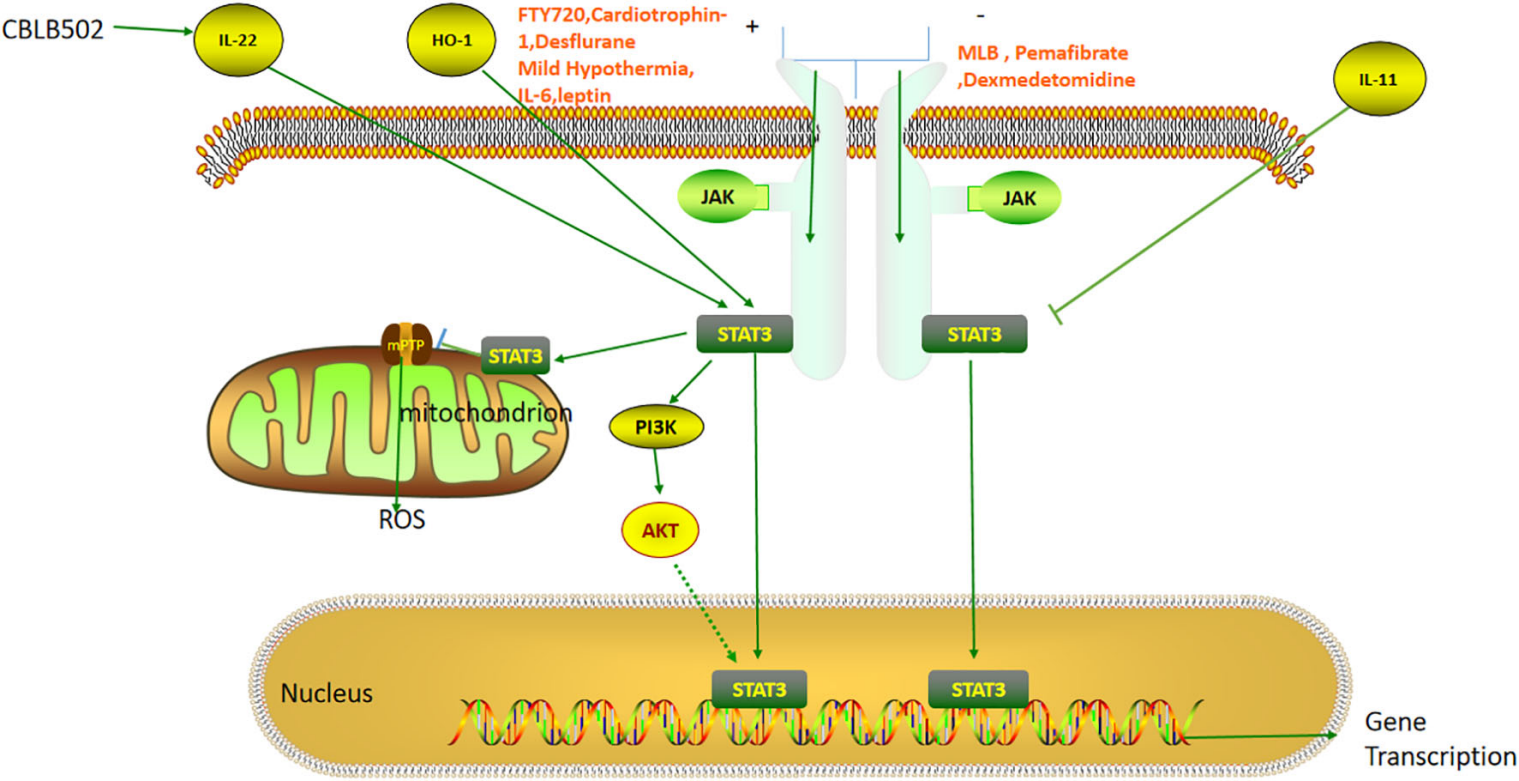
The role of the STAT3 pathway in liver ischemia-reperfusion injury. FTY720, Cardiotrophin-1, Desflurane, Mild Hypothermia, IL-6, and Leptin reduce hepatic ischemia-reperfusion injury by activating JAK-STAT3 pathway. MLB, Pemafibrate, and Dexmedetomidine can attenuate the injury by inhibiting the JAK-STAT3 pathway. IL-22, HO-1 and IL-11 can directly activate and inhibit STAT3 respectively. STAT3 can also attenuate ischemia-reperfusion injury by inhibiting mPTP opening and thereby reducing ROS release.

The JAK-STAT pathway was originally discovered by Darnell et al. It is known to be an important intracellular signal transduction pathway, and has been implicated in the regulation of growth, differentiation, apoptosis, and development of various cells (**Figure 3**). It can promote the phosphorylation and activation of diverse proteins with tyrosine residues, generate a cascade reaction of kinase activation, and transduce the activated signal to other molecules, such as STAT, thereby triggering a series of genetic and protein changes (75, 76). In mammals, more than 40 cytokines can potentially activate the JAK -STAT pathway to influence hepatic IRI (77–79). JAK is a tyrosine kinase widely present in different types of cells, and can be divided into four types, namely, JAK1, JAK2, JAK3, and TYK2 (80). When the ligand binds to its surface receptor, it causes changes in the cytosolic part of the receptor, thus promoting the phosphorylation of the JAK protein. STAT3 and JAK are closely bound together in the resting state. When JAK is phosphorylated, STAT3 and JAK are separated to promote the phosphorylation of STAT3, and the activated STAT3 can undergo heterodimerization followed by the translocation to the nucleus, which can promote the transcriptional expression of the various genes (80–82). The STAT3 TAD contains three different sites, namely Y701, Y705, and Ser 727. Since TAD contains binding sites for its own dimer, it is quickly activated once stimulated in the cell. Exposure of Tyr705 can accelerate the process of heterodimerization, and it has been documented that increased phosphorylation of STAT3 at Tyr705 in the nucleus can reverse the inhibition of STAT3 phosphorylation at 727 during IRI (20, 83, 84).

JAK-STAT3 activation can attenuate liver IRI. For instance, Mahmoud AR has found that coenzyme Q10 (CoQ10), which forms part of the mitochondrial respiratory chain in hepatocytes, was able to suppress apoptosis and oxidative stress by activating the JAK1/STAT3 pathway (85). Fingolimod (FTY720) is an inhibitor of the Sphingosine-1-phosphate (S1P) receptor with diverse anti-inflammatory effects (86). Xiangmin He demonstrated that Fingolimod (FTY720) could activate the JAK2/STAT3 pathway, thereby inhibiting hepatic IRI induced by acetaminophen (APAP) (87). Relevant studies have confirmed that STAT3 in hepatocytes can promote liver regeneration after hepatectomy. However, Feng D found that STAT3 had no effect after 6 h of APAP-induced ALI (88, 89) while Nishina T observed that STAT3 was still functional after 24 h; thus the specific relationship between APAP and STAT3 requires further investigation (90).The authors also reported that P-JAK2/P-STAT3 expression decreased after ischemia-reperfusion alone, which was inconsistent with previous results. The authors explained that it was related to time, and the times of IRI in this model were 1 h and 6 h, both of which were significantly shorter than those used in previous studies (91). Therefore, it is necessary to further study the activation of the pathway at the different time points in the future. Heng Chao Yu found that the Notch pathway could also activate JAK2/STAT3, promote the expression of manganese superoxide dismutase (MnSOD), inhibit ROS and apoptosis, and ultimately attenuate hepatic IRI (92). Cardiotrophin-1 was originally used as a drug to promote cardiac hypertrophy (93). Maria Iniguez reported that the myocardial nutrient cardiotrophin-1 could attenuate hepatic IPI by activating the JAK/STAT3 pathway, but the specific mechanism requires further study (94). Mengxia Zhong found that desflurane inhibited mir-135b-5p, thereby stimulating JAK2-STAT3 activation, inhibiting apoptosis, and ultimately alleviating liver IRI (95). However, there are also several reports that support the opposite conclusion. L Xiong found that mir-93 could inhibit the JAK/STAT3 pathway, thereby suppressing the production of apoptosis, inflammatory factors and transaminases, and leading to the alleviation of liver IRI (96). Ziqi Cheng reported that pemafibrate, a selective inhibitor of PPARα, could inhibit the release of inflammatory factors produced by Kupffer cells, attenuate the JAK2/STAT3β/PPARα pathway, suppress cell apoptosis as well as autophagy, and ultimately attenuate liver IRI (97). Ning Zhang found that Magnesium Lithospermate B, a traditional Chinese medicine, could markedly inhibit the production of inflammatory factors such as TNF-α and IL-6 by inhibiting the JAK2/STAT3 pathway, thereby attenuating hepatic IRI (98). Maria Cecilia S. Freitas reported that the JAK2 inhibitor AG490 could negatively regulate JAK-STAT signaling, thereby reducing the production of inflammatory factors, inhibiting apoptosis, and ultimately reducing IRI. It was also found that STAT1 activation was more likely to cause IRI than STAT3 (99). Y X Zhu found that dexmedetomidine can inhibit JAK/STAT3 signaling, apoptosis and the inflammatory response, as well as oxygen-glucose deprivation (OGD)-mediated human hepatic IRI (100). STAT1 and STAT3 have been reported to exert opposite effects on cell proliferation, differentiation, and apoptosis, which deserves further study in the future (101).

## 6 STAT3 and PI3K/AKT

The phosphoinositide-3 kinase (PI3K)/protein kinase B (PKB/AKT) is an important pathway involved in protein synthesis and is closely related to the regulation of redox reactions in mitochondria. PI3K/AKT can alleviate hepatic IRI by inhibiting the release of inflammatory factors and cell apoptosis while promoting autophagy (102). Bibo Ke reported that heme oxygenase-1 (HO-1) could promote the phosphorylation of STAT3, activate PI3K/AKT, inhibit the release of TNF-α and IL-10 induced by TLR-4, leading to reduced IRI (103). Bibo Ke found that STAT3 could activate PI3K/AKT by activating β-catenin, inhibiting IL-12 and Bax, and ultimately attenuating liver IRI (104). Huang J found that the nuclear factor E2-related factor 2 (Nrf2)-HO-1 axis could activate the Notch1/Hairy and enhancer of split homolog-1(Hes1)/STAT3 pathway, promote the macrophage differentiation and PI3K/AKT pathway activation, inhibit apoptosis, and ultimately reduce liver IRI (105). Therefore, the STAT3/PI3K/AKT pathway can play a key role in acute injury but the specific downstream mechanism of PI3K/AKT needs to be further explored.

## 7 STAT3 and lipid metabolism

**Figure 4:**
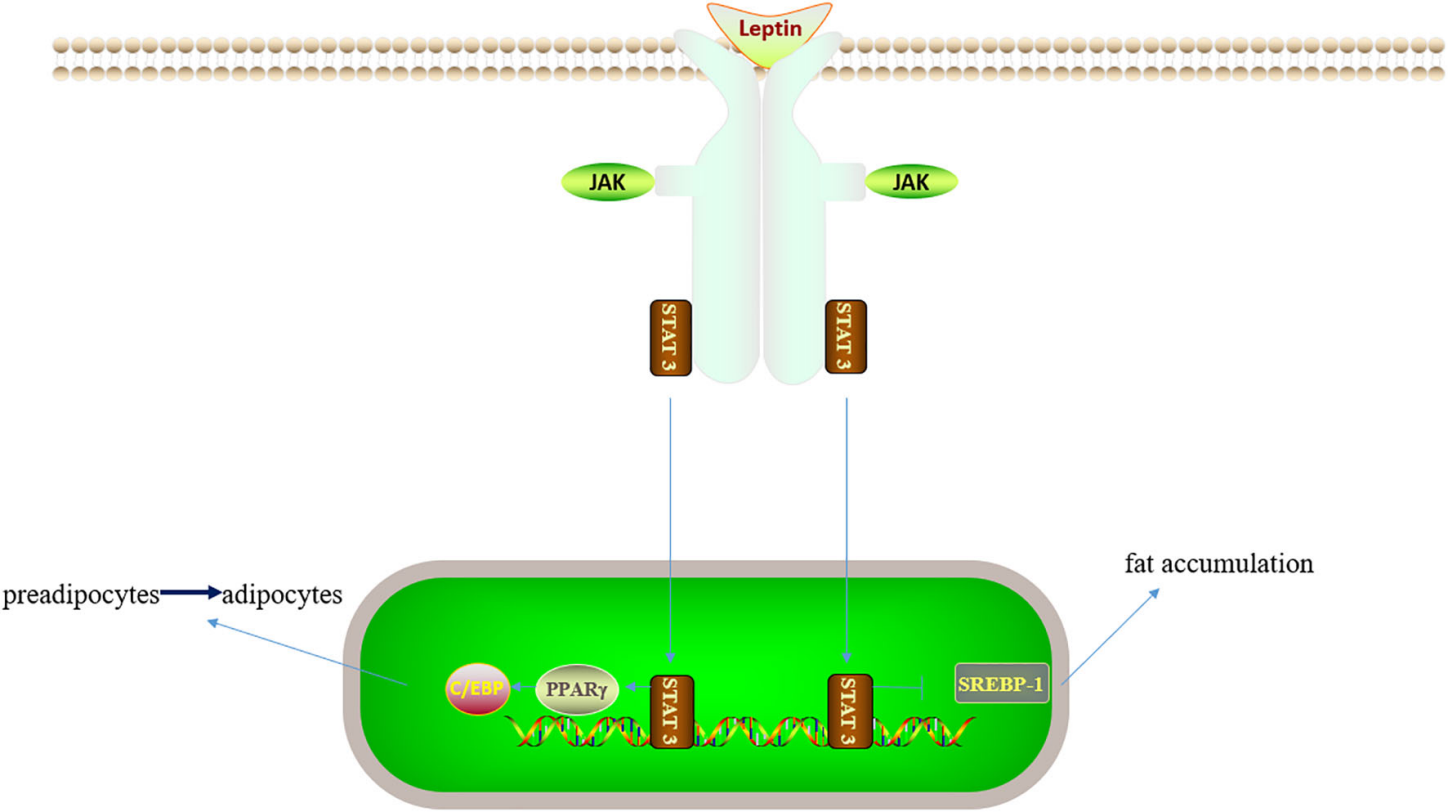
Leptin/JAK/STAT3 related lipid metabolic pathway. On the one hand, STAT3 can activate PPARγ, C/EBP and inhibit liver adipocyte maturation; on the other hand, STAT3 can inhibit SREBP-1 and thus inhibit fat accumulation. Therefore, STAT3 has different roles in lipid metabolism.

Given the increasing number of patients with nonalcoholic fatty liver disease (NAFLD), the number of patients with fatty liver disease requiring organ transplantation has increased. Fatty liver is susceptible to IRI and two different hypotheses have been proposed to account for this, namely, impaired hepatic microcirculation and mitochondrial dysfunction (**Figure 4**). The volume of the steatotic hepatocytes becomes larger, squeezing and narrowing the perisinusoidal space, thus increasing the resistance of hepatic microcirculation. Fatty liver can also cause mitochondrial dysfunction through promoting the production of ROS, thereby interfering with cellular energy metabolism in the liver (106, 107). STAT3 activates peroxisome proliferator-activated receptor (PPAR)γ, then up-regulates the transcription of C/EBP, and promotes the transformation of preadipocytes into adipocytes. STAT3 knockout mice showed weight gain due to hypertrophy of adipocytes, suggesting that STAT3 plays a role in lipid degradation. JAK2-STAT3 promotes lipid degradation by inhibiting the expression of fatty acid synthase and acetyl-CoA carboxylase (108, 109). In several adipose models, STAT3 knockdown in the hepatocytes was observed to aggravate steatosis (35, 110, 111). Sterol regulatory element-binding protein-1 (SREBP-1) is a transcription factor that can regulate liver lipid metabolism. STAT3 can inhibit hepatic fat accumulation by suppressing SREBP-1, and ultimately reduce hepatic steatosis (110, 112). Marco Carbone reported that the addition of leptin to the preservation solution could activate STAT3 and reduce the degree of apoptosis, thereby attenuating the development of cold IRI (113). However, the specific mechanism of STAT3 in liver cold preservation needs to be further explored. Renalase is a ubiquitous pan-xanthine dinucleotide amine oxidase found in many organs (114). Tao Zhang reported that renalase could activate the STAT3-SIRT1 pathway and inhibit IRI in fatty liver (17). Zhihui Jiao found that the secretory proteome of adipose-derived mesenchymal stem cells could inhibit the expression of SOC3 and the negative feedback effect of SOC3 on STAT3 can lead to increase the expression of P-STAT3, and reduce IL-6, TNF-α and other related inflammatory factors, thereby alleviating liver IRI (115). Euno Choi found that P-STAT3 might aggravate liver steatosis and inflammatory injury, which was the first time for P-STAT3 to be explored in specimens of patients with fatty liver disease. The authors did not clarify whether this effect was related to the leptin pathway, and further research is needed to explore the relationship (116).Therefore, STAT3 plays different roles in regulating the process of lipid metabolism, and future studies to investigate its role in fatty liver are warranted.

## 8 Conclusion

In hepatic IRI, STAT3 usually binds to the mitochondria to regulate programmed cell death. STAT3 plays a role in many hepatic cells. The STAT3-associated IR pathway includes upstream cytokines, and JAK, and downstream PI3K/AKT. The role of STAT3 in liver IRI is controversial (**Table 1**). On the one hand, STAT3 can play a protective role through the modulation of various proteins, inflammatory factors, and cells while on the other hand, it can aggravate IRI. The reason can be partly attributed to the fact that that P-Janus kinase (P-JAK) can activate both STAT3 and STAT1, and STAT3 can inhibit apoptosis whereas STAT1 can promote apoptosis. The JAK-specific inhibitor AG490 can inhibit both, thus producing different effects, but these are closely related to the length of the model time (117). The same protein may have different effects at different times and the same inflammatory factors can play diverse roles. STAT3 can also play a dual role in the regulation of lipid metabolism. On the one hand, STAT3 can promote the maturation of adipocytes while, on the other hand, it can promote lipolysis. STAT3 may also affect the microcirculation and energy metabolism by influencing fat accumulation. Therefore, STAT3 has an important effect on IRI in fatty liver. The incidence of fatty liver is increasing and it is necessary to further explore the functions of STAT3 in adipocyte maturation and lipolysis. Thus, further analysis of STAT3-related pathways in liver IRI is needed to provide a foundation for clinical treatment.

**Table 1:** An overview of the role of STAT3 in hepatic ischemia-reperfusion.

Author	Journal	Year	Species	Finding
Iñiguez M	J Exp Med	2006	mouse	Cardiotrophin-1 alleviates hepatic IRI by activating the JAK/STAT3 pathway.
Yu HC	Hepatology	2011	mouse	The Notch pathway can activate the JAK2/STAT3 pathway, promote the expression of manganese superoxide dismutase (MnSOD), ultimately alleviate hepatic IRI.
Ke B	J Hepatol	2012	mouse	HO-1 promotes STAT3 phosphorylation, activating PI3K/AKT thereby alleviating liver IRI.
Carbone TM	Transpl Int	2012	rat	Leptin can activate STAT3 and reduce the degree of necrosis and apoptosis, thereby alleviating cold IRI.
Chestovich PJ	Transplantation	2012	mouse	IL-22 can promote the phosphorylation of STAT3, inhibit the production of inflammatory factors, and ultimately alleviate liver IRI.
Ke B	Hepatology	2013	mouse	STAT3 can activate β-catenin followed by activation of PI3K/AKT, and ultimately alleviate hepatic IRI.
Huang J	Mol Med	2014	mouse	The Notch1/Hes1/Stat3 pathway promotes the activation of PI3K/AKT pathway, ultimately alleviating liver IRI.
Zhu M	PLoS One	2015	mouse	IL-11 can inhibit the phosphorylation of STAT3, thereby inhibiting the activation of inflammatory factors, and ultimately alleviating liver IRI.
Li S	Liver Transpl	2016	mouse	mir-17 promotes the expression of LC3BII by inhibiting the expression of STAT3, and ultimately aggravates hepatic IRI.
Rao Z	Front Immunol	2017	mouse	Hyperglycemia can inhibit STAT3 through C/EBP protein mediated ER stress, thus aggravating liver IRI.
Han YF	J Cell Biochem	2018	mouse	STAT3 can activate ATG5 protein-mediated autophagy, thereby alleviating IRI.
Zhu YX	Eur Rev Med Pharmacol Sci	2018	human	Dexmedetomidine can inhibit the JAK/STAT3 pathway, and ultimately inhibit oxygen-glucose deprivation (OGD) -mediated IRI.
Mahmoud AR	Tissue Cell	2019	rat	CoQ10 inhibits apoptosis and oxidative stress by activating the JAK1/STAT3 pathway.
Xie K	IUBMB Life	2019	mouse	Mir-1246 regulates IL-6-GP130 -STAT3 axis, and ultimately alleviates hepatic IRI.
Zhang N	Front Pharmacol	2019	mouse	MLB could inhibit the JAK2/STAT3 pathway, thus inhibiting liver IRI.
Zhang T	Oxid Med Cell Longev	2019	mouse	Renalase can activate the STAT3-SIRT1 pathway and inhibit IRI of fatty liver.
Zhu YX	Int Immunopharmacol	2020	mouse	Dexmedetomidine can activate the PPARγ/STAT3 pathway, ultimately alleviating liver IRI.
Zheng L	J Immunol	2020	mouse	Roquin 1 inhibits the activity of AMPKa and promotes the activation of mTOR and STAT3, thereby alleviating liver IRI.
Wang W	Oxid Med Cell Longev	2020	mouse	Mild hypoxia was found to activate the JAK2-STAT3-CPT1A pathway, ultimately promoting the β -oxidation of fatty acids, and ultimately alleviating liver IRI.
Ozturk A	FASEB Bioadv	2020	human	BWP1066 inhibits the release of inflammatory cytokines from macrophages thereby alleviating hepatic IRI.
Shang L	Oxid Med Cell Longev	2021	mouse	SS-31 can inhibit the STAT3, ultimately alleviating liver IRI.
Zhong M	J Chin Med Assoc	2021	rat	Desflurane can inhibit mir-135b-5p to promote the activation of JAK2-STAT3, ultimately alleviating liver IRI.
Melin N	Cell Death Dis	2021	mouse	CBLB502 alleviates hepatic IRI through the IL-22-STAT3 pathway.
Jiao Z	Stem Cell Res Ther	2021	pig	The adipose-derived mesenchymal stem cell secretome inhibit the expression of SOC3, increase the expression of P-STAT3, to alleviate liver IRI.
Zhou H	J Immunol Res	2021	mouse	IL-22 can promote the phosphorylation of STAT3, which in turn promotes the expression of cyclinD1, and ultimately alleviates liver IRI.
Cheng Z	PPAR Res	2021	mouse	Pemafibrate can inhibit JAK2/STAT3β/PPARα pathway, ultimately alleviating liver IRI.
Tang H	Mol Biol Rep	2021	human	Recombinant human IL-6 can activate STAT3, inhibit autophagy proteins, and ultimately alleviate liver IRI.
He X	Int J Mol Med	2022	mouse	FTY720 activates the JAK2/STAT3 pathway to inhibit hepatic IRI induced by APAP.
Li W	Peer J	2022	mouse	Ac2-26 can protect ATP, mitochondrial membrane potential (MMP), and ultimately reduce IRI.

## Author contributions

HY, YZ, PZ contributed to manuscript writing and editing. HY, YZ, PZ, QW, KC conducted a critical revision of the manuscript. All authors contributed to the article and approved the submitted version.
